# Supplementary material for: Myocardin-related transcription factor regulates actomyosin contractility and apical junction remodeling during vertebrate neural tube closure
Source: Development. 2025 Aug 26;152(16):dev204681. doi: 10.1242/dev.204681 (PMC12448316; doi:10.1242/dev.204681)
Supplement: Supplementary information [file develop-152-204681-s1.pdf]

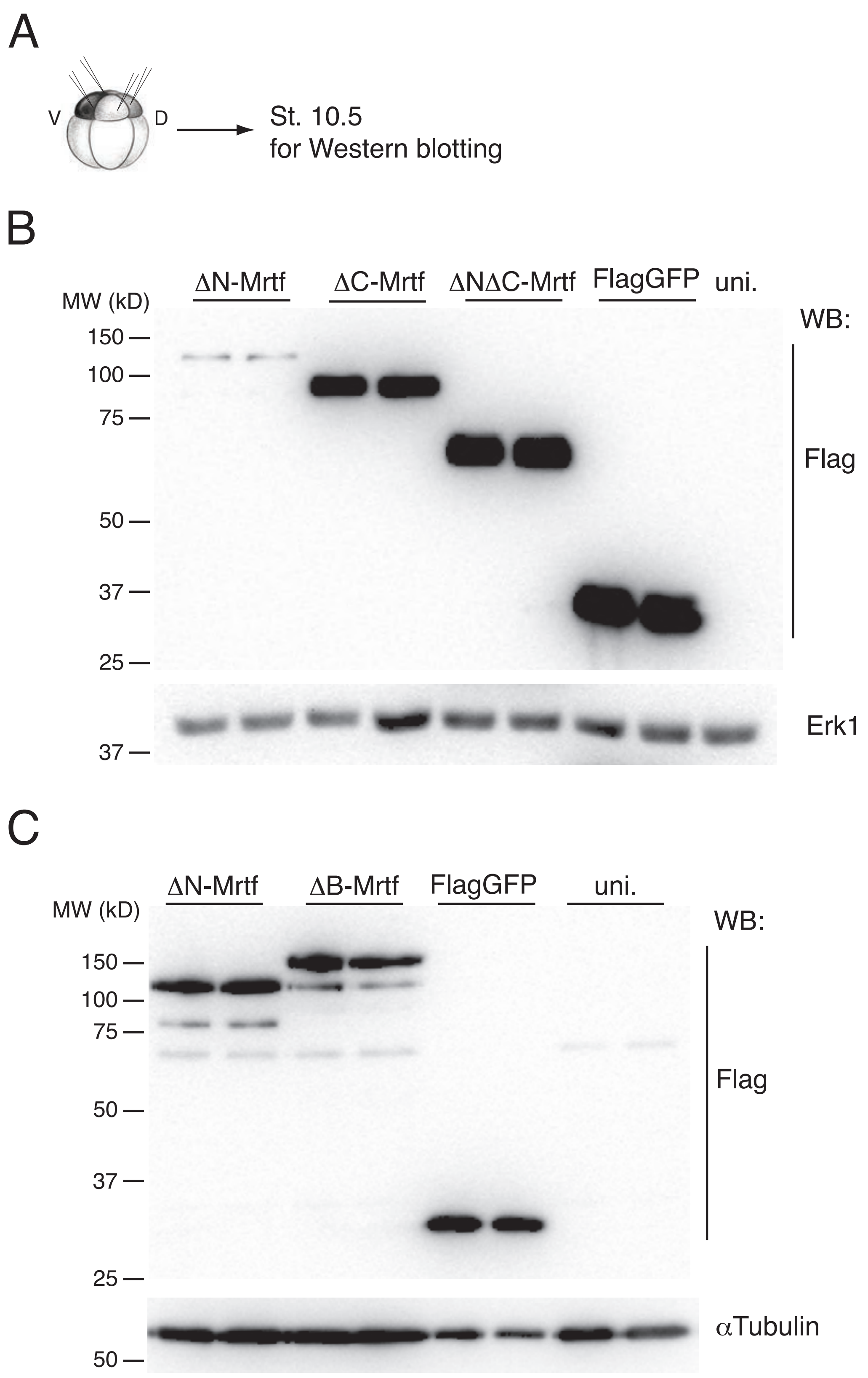

Fig. S1. Expression levels of Mrtfa constructs.

A, Experimental scheme. Four animal blastomeres at 4-8 cell stages were injected with RNAs indicated in B and C. When control embryos reached stage 10.5, injected embryos were lysed for immunoblotting. B, Expression levels of Mrtf constructs in *Xenopus* embryos. Injected RNAs, ΔN-Mrtf (500 pg), ΔC-Mrtf (500 pg), ΔNΔC-Mrtf (500 pg) or FlagGFP (500 pg). Immunoblotting with anti-Flag and anti-Erk1 antibodies is shown. Erk1 serves as a loading control. C, Expression levels of Mrtf constructs in *Xenopus* embryos. Injected RNAs, ΔN-Mrtf (250 pg), ΔB-Mrtf (250 pg) or FlagGFP (250 pg). Immunoblotting with anti-Flag and anti-αTubulin antibodies is shown. For FlagGFP, one fifth of the samples was loaded. αTubulin serves as a loading control.

Development • Supplementary information

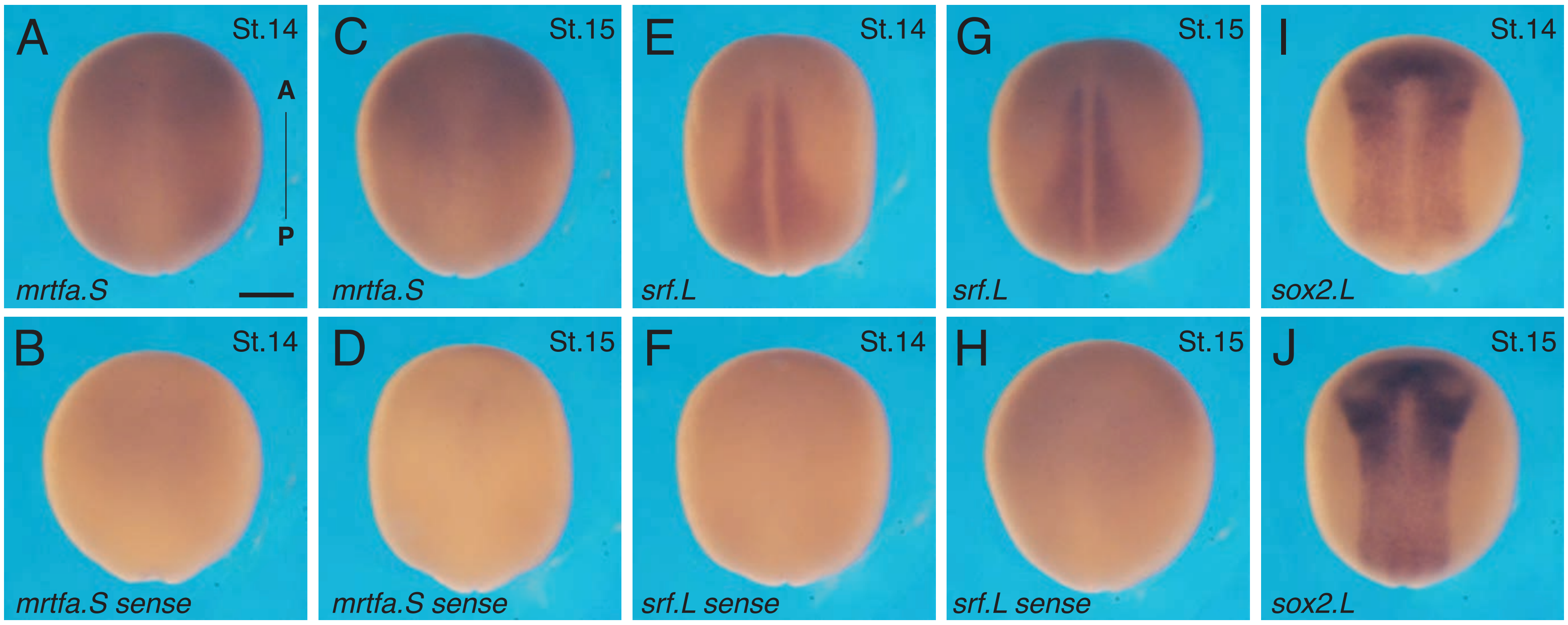

Fig. S2. Expression of Mrtfa and SRF mRNAs at neurula stages.

Whole mount in situ hybridization was carried out with anti-sense and sense RNA probes of *Mrtfa.S* (A-D) and *SRF.L* (E-H) genes at stage 14 and 15. I, J, Neural plate is marked with *Sox2.L* probe. Dorsal view with anterior top (A) and posterior bottom (P) is shown in all images. Scale bar: 300 μm.

Development • Supplementary information

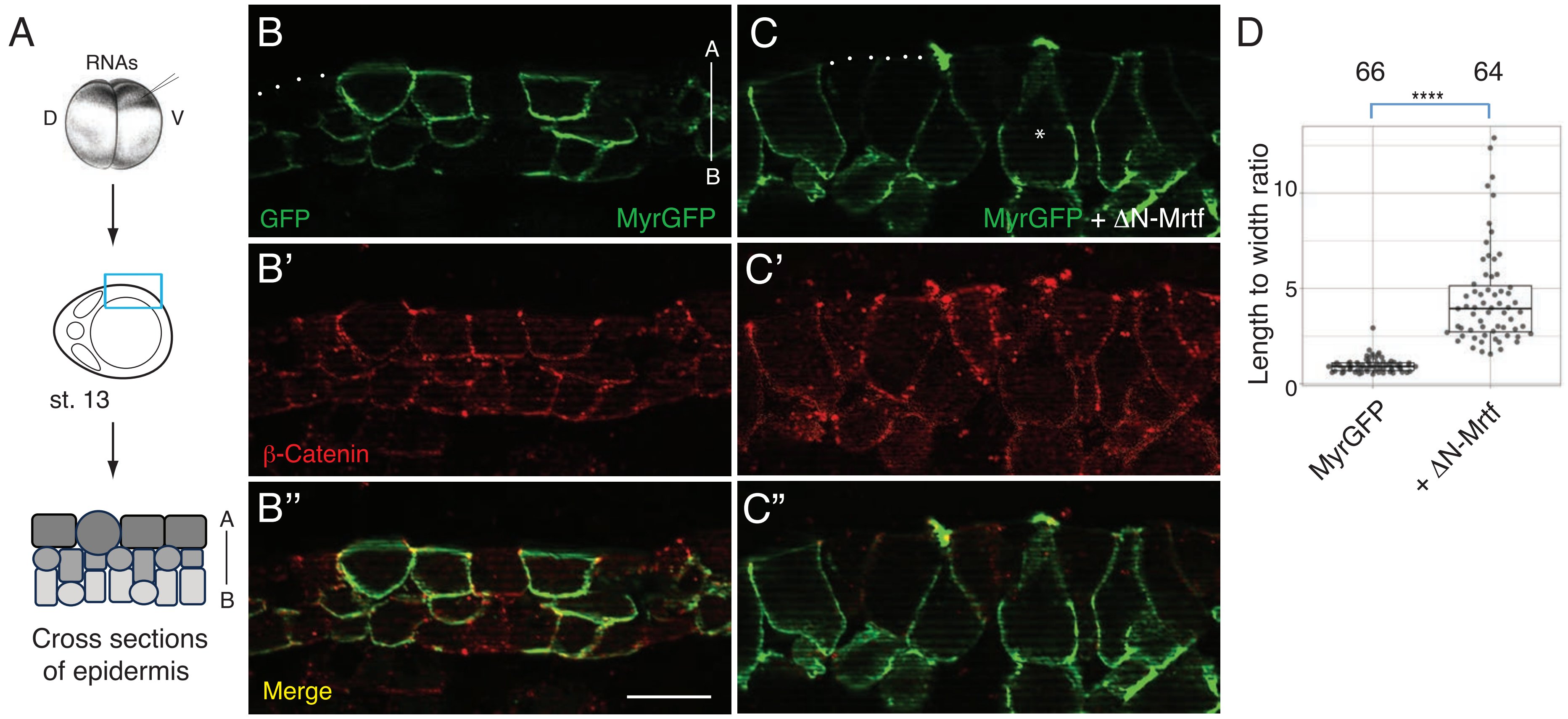

Fig. S3. Bottle cell morphology of ΔN-Mrtf expressing cells.

A. Experimental scheme. Embryos were injected with 250 pg of ΔN-Mrtf RNA and 25 pg of MyrGFP RNA as indicated in Fig. 4 legend. MyrGFP is a lineage tracer. B, C, Transverse cryosections of control (B) or ΔN-Mrtf-expressing (C) ventral ectoderm (stage 13) immunostained for GFP and β-catenin. Punctate lines indicate apical cell surface. Scale bar, 20 μm. D. Quantification of the ratio between cell length along the apical-basal axis and apical domain width of superficial ectodermal cells. Numbers of scored cells are indicated on top. Mann Whitney test. ****p<0.0001.

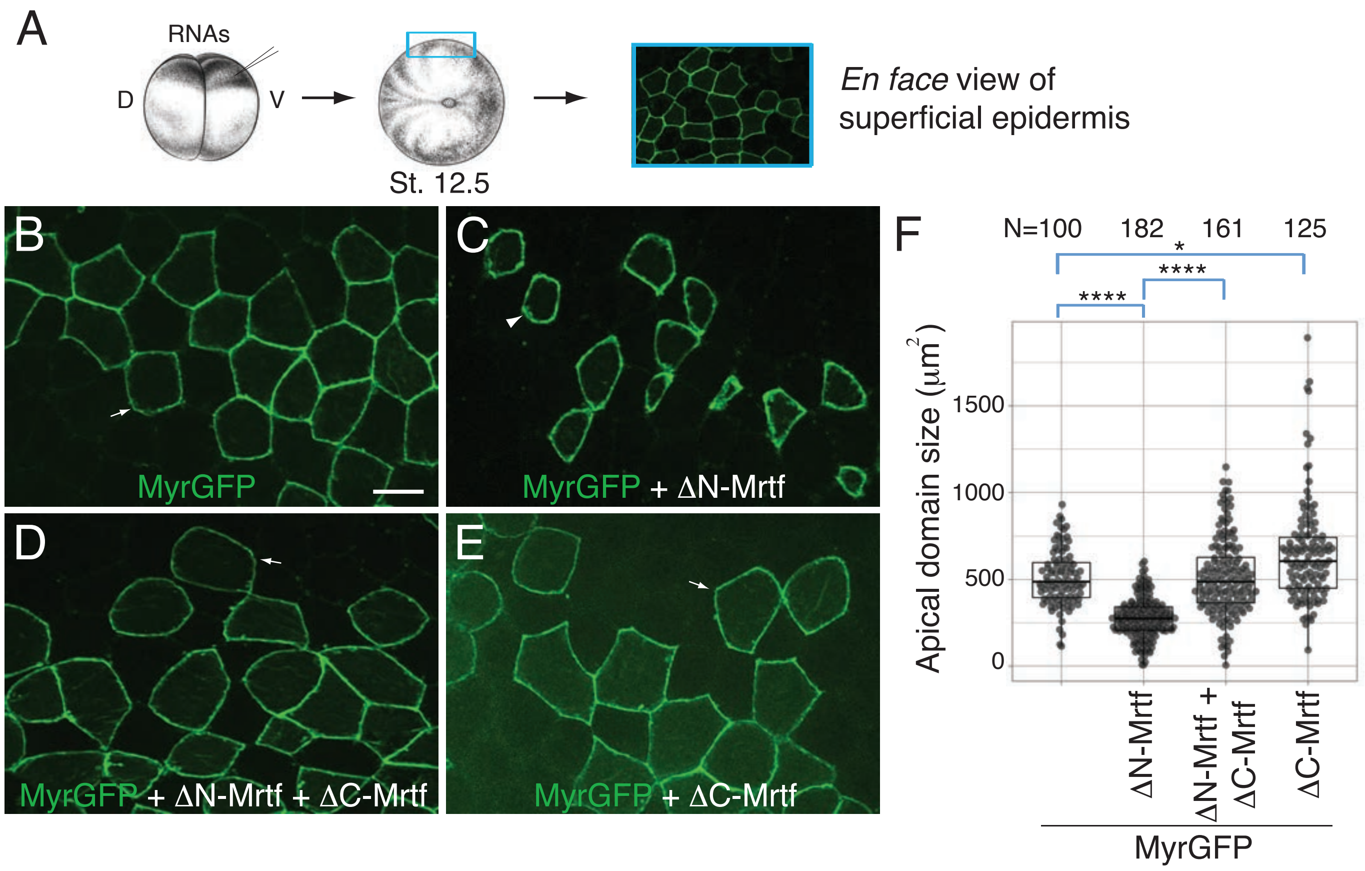

Fig. S4. Mrtfa lacking transcription activation domain inhibits Mrtfa-induced apical constriction.

A. Experimental scheme. Four-cell embryos were injected into one-ventro-animal blastomere with MyrGFP RNA (50 pg) alone (B) or with 250 pg ΔN-Mrtf RNA (C), with 250 pg ΔN-Mrtf and 1 ng ΔC-Mrtf RNAs (D) or with 1 ng ΔC-Mrtf RNA (E). Superficial epidermal ectoderm of the injected embryos at stage 12.5 was imaged. Arrows (B, D, E) indicate mosaic cells. Arrowhead in C indicates an example of reduced size of a mosaic cell upon ΔN-Mrtf expression. Scale bar, 20 μm. F. Apical domain size of mosaic GFP expressing cells in B-E was measured. Numbers of scored cells are on the top. One-way ANOVA Kruskal-Wallis test. *p<0.05, ****p<0.0001. Scale bar, 20 μm.

Development • Supplementary information

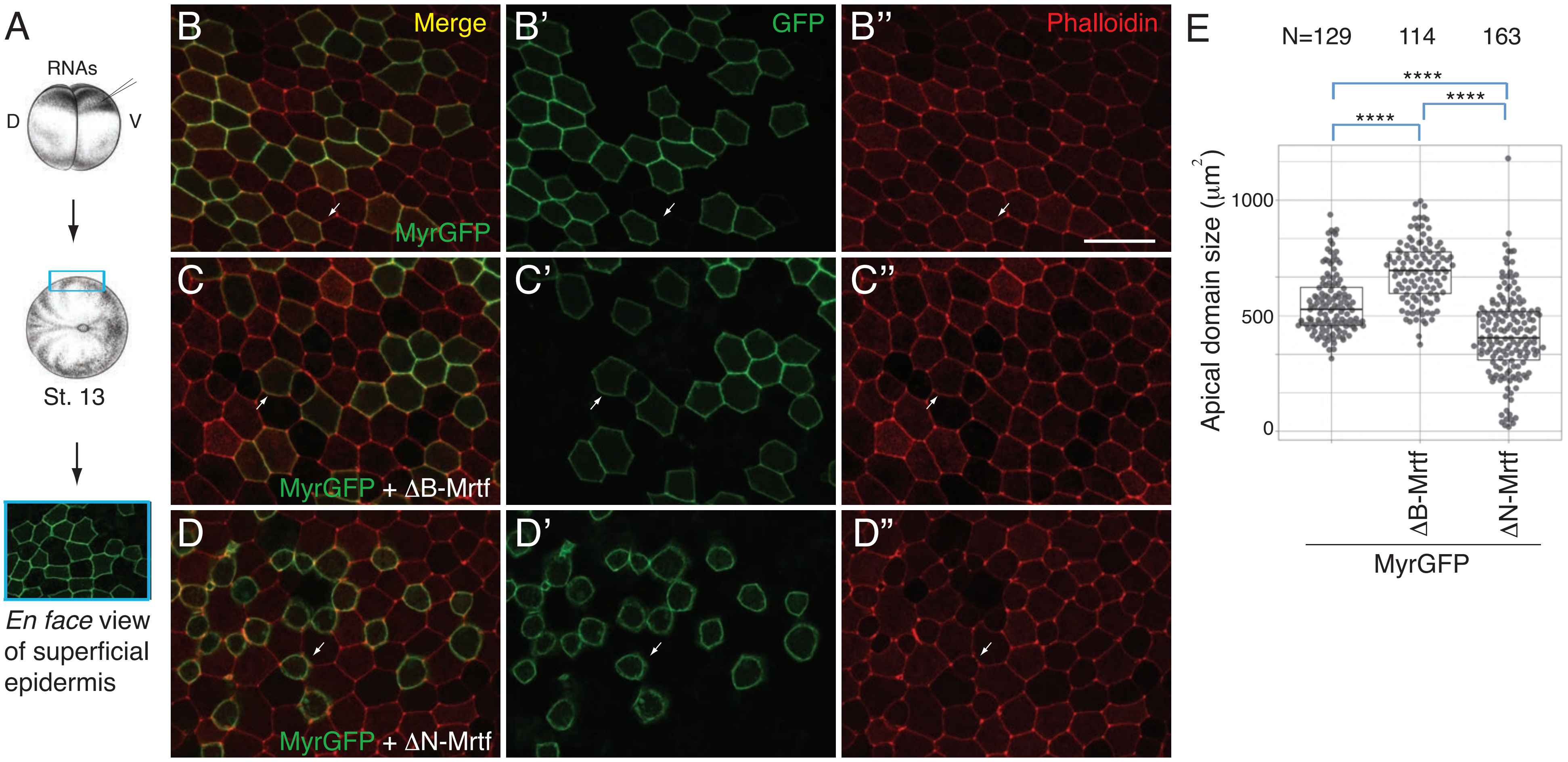

Fig. S5. Mrtfa lacking SRF binding domain does not induce apical constriction in non-neural ectoderm.
A. Experimental scheme. Four-cell embryos were injected into one ventral-animal blastomere with MyrGFP RNA (25 pg) alone (B) or with 250 pg ΔB-Mrtf RNA (C), or with 250 pg ΔN-Mrtf RNA (D). Superficial epidermal ectoderm of the injected embryos at stage 13 was imaged after phalloidin staining (B-D). Arrows point to the cells mosaically expressing GFP. Scale bar: 50 μm. E. Apical domain size of mosaic cells was measured. Numbers of scored cells are on the top. Kruskal-Wallis test, ****p<0.0001.

Table S1. List of primer sequences used for RT-PCR and RT-qPCR.

Primers used for RT-PCR

eef1a1.L F: 5’-CAGATTGGTGCTGGATATGC-3’
R: 5’-ACTGCCTTGATGACTCCTAG-3’

mrtfa.L F: 5’-CTCAATGGGATCCACGTG-3’
R: 5’-GCTTTACTAGTGCTGGCA-3’

Primers used for RT-qPCR

eef1a1.S F: 5’-ACCCTCCTCTTGGTCGTTTT-3’
R: 5’-TTTGGTTTTCGCTGCTTTCT-3’

acta2.S F: 5’-CAATTGGAAATGAGCGTTTC-3’
R: 5’-CATCTGCTGGAAGGTAGACA-3’

actc1.L F: 5’-CGGTATCCATGAAACTACCT-3’
R: 5’-CGGAGTATTTACGCTCAGGT-3’

myl3.L F: 5’-TCTTGGCTACTCTAGGGG-3’
R: 5’-ATTTCCGACCACATTACCTT-3’

Development • Supplementary information

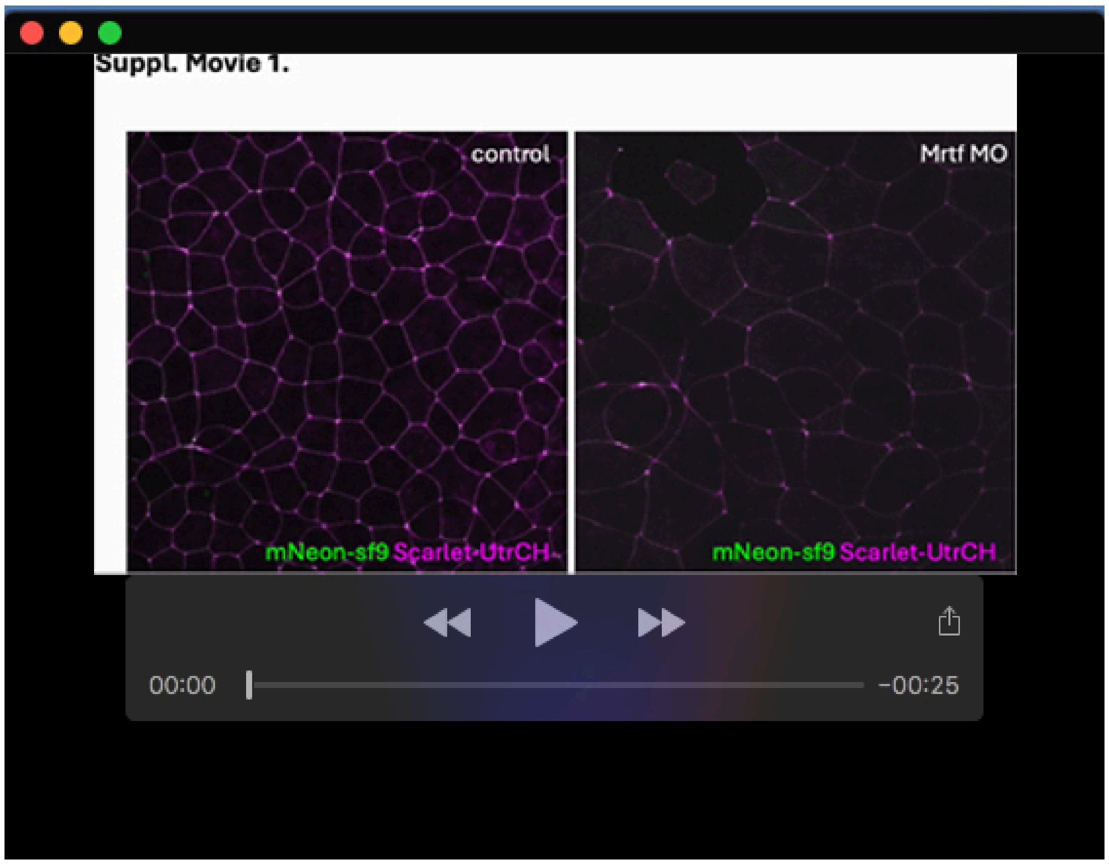

Movie 1. Time-lapse live imaging of superficial ectoderm cells from stage 11 embryos using Andor BC43 confocal microscope (20 x objective lens). 4-8-cell embryos were coinjected with MrtfaMO (40 ng), mNeonGreen-Sf9 (green) and Scarlet-UtrCH (magenta) RNAs (50-100 pg each). Length of the movie 60 min with 3 min intervals.

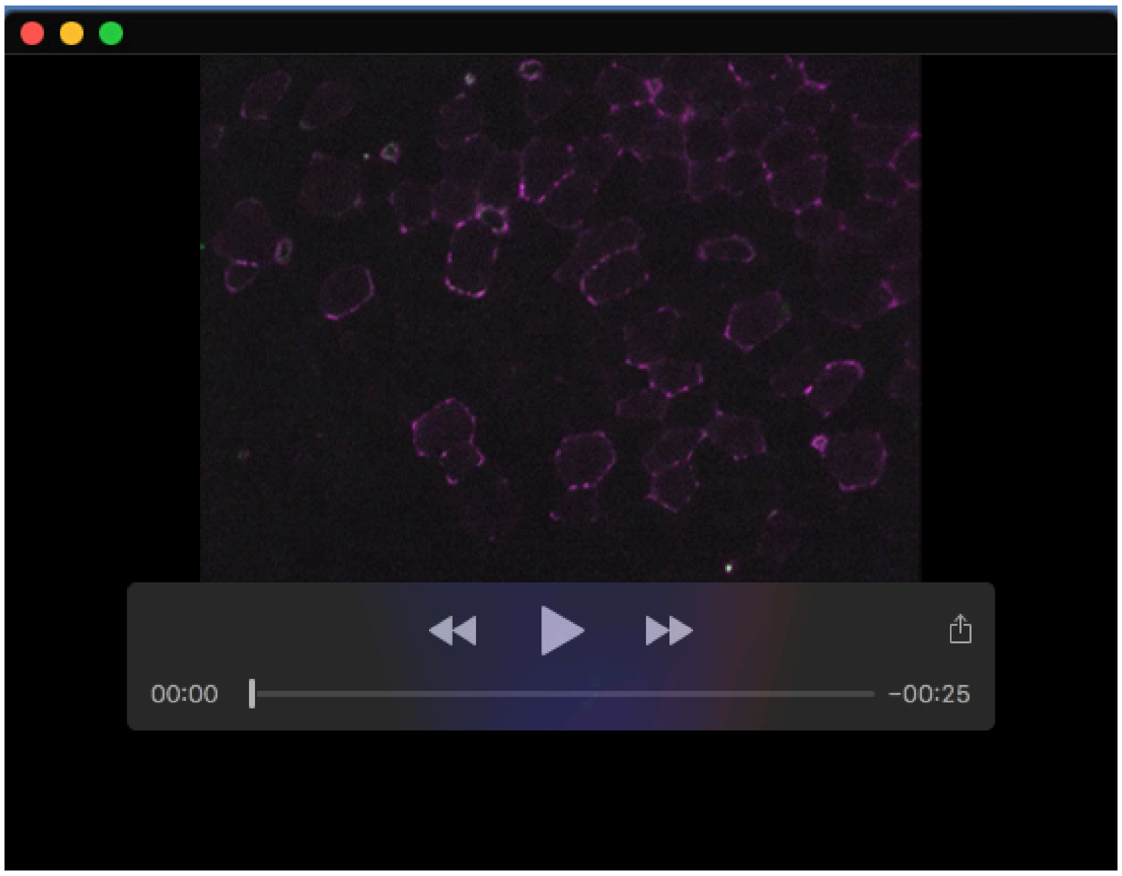

Movie 2. Time-lapse live imaging of mosaically expressing ventral ectoderm from stage 13 embryos using Andor BC43 confocal microscope (20 x objective lens). Four-cell embryos were coinjected with 50-100 pg of RNAs encoding ΔN-Mrtf (250 pg), mNeonGreen-Sf9 (green, 50 pg) and Scarlet-UtrCH (magenta, 50 pg). Top view shows apical domain reduction in several cells as compared to apical domain size of their neighbors. Length of the movie 60 min with 3 min intervals.

Development • Supplementary information
